# Ascertaining social worker contacts in routine mental healthcare and describing their distribution: a descriptive analysis of electronic records data from a large south London mental healthcare provider

**DOI:** 10.1136/bmjopen-2024-090055

**Published:** 2025-03-26

**Authors:** Norah Alothman, Amelia Jewell, Gayan Perera, Robert Stewart

**Affiliations:** 1 Department of Psychological Medicine, Institute of Psychiatry Psychology & Neuroscience, King’s College London, London, UK; 2 NIHR Maudsley Biomedical Research Centre (BRC), South London and Maudsley NHS Foundation Trust, London, UK

**Keywords:** MENTAL HEALTH, Health policy, Health Workforce, Social Support

## Abstract

**Abstract:**

**Objectives:**

To describe the distribution of contacts with mental health service-employed social workers over time and by patient characteristics using routine mental health service data resources.

**Design:**

A descriptive study.

**Setting and participants:**

In a large secondary mental healthcare provider in London serving a geographic catchment of around 1.3 million residents, mental health social worker contacts were ascertained from the case note entries for all patients aged 16 years or above at the time of contact who received treatment in any services from 2008 to 2023.

**Main outcome measures:**

Patient demographic and clinical characteristics at or closest to the social worker contact event.

**Results:**

A total 1 541 078 social worker contacts were extracted. Contacts were most likely in the 20–39 years age group (38.1%), in men (51.9%), in patients from white (45.3%) and black (38.8%) ethnic groups, in those who were non-cohabiting (89.9%) and in those living in more deprived neighbourhoods. The most likely diagnosis in those receiving social work contacts was schizophrenia (39.2%). Males had the highest number of face-to-face social worker contacts, and females were more represented in phone and video contacts. Over the past 16 years, social worker contacts were highest between 2014 and 2015.

**Conclusions:**

To the best of our knowledge, this study is the first quantification of social work deployment within mental healthcare. Research into the role of social workers within mental health services has been of small scale and predominantly qualitative to date. However, growing data resources, building on distributions of service provision, present important opportunities for wider evaluation of the role of this professional group and the interventions they support within multidisciplinary teams.

**STRENGTHS AND LIMITATIONS OF THIS STUDY**

- Our study employed a very large real-world sample from diverse services to investigate social work involvement in mental healthcare.
- The study should have near-complete ascertainment of mental healthcare-employed social worker contacts to the extent that these were recorded in case notes.
- Despite the large amount of data from diverse services and a highly diverse source population, findings from this study are drawn from only one site and would require further investigation for generalisability.
- This descriptive analysis did not attempt to capture the level and intensity of involvement, which requires further investigation.
- Social work inputs were captured from mental health service staff; in this approach, we did not attempt to capture inputs from social workers employed externally.

## Background

 Social workers provide a core component of mental healthcare in the United Kingdom and internationally.^1^ In England, there were an estimated 100 654 registered social workers in 2022 (17 762 in London alone), approximately 1 per 500 residents.^2^ They work in a range of settings, including hospital and community mental health services, and with different age groups (children, adolescents and adults) to deliver care directly and in case management.^3^ Social workers’ roles are crucial in mental healthcare, as they have skills and knowledge that particularly suit the practice of mental health intervention,^4^ contributing to ensuring that services meet patients’ needs as well as promoting recovery and supporting individuals to live independently.^5^ Social workers also have a long history of working with individuals, families and society, dealing with many aspects to ensure that appropriate support is provided.

In England, social work, in common with other professions, has experienced changes over recent decades. These have included the Mental Health Act of 1959, which assigned social workers new duties in medicolegal aspects of mental healthcare, developing into the Approved Social Worker role instigated in 1983 before this was widened in 2007 to Approved Mental Health Professionals (AMHPs). AMHPs have specific responsibilities, including organising statutory mental health assessments, communicating with the required agencies and nearest relatives. The main principle is to support the human rights of individuals assessed under the Mental Health Act.^6^ AMHPs can come from a range of professions, including nurses, occupational therapists and psychologists; however, a recent AMHP workforce survey found that 94% in England were social workers (93% for London specifically).^7 8^ In addition, social workers also comprised the majority of Best Interest Assessors, a role created in 2009 under the Mental Health Capacity Act to assess whether a person has mental capacity and is being deprived of liberty for their treatment, subsequently replaced by the Approved Mental Capacity Professional role.^2 9^

Furthermore, social workers also work in collaboration with professionals from other sectors, such as housing and employment specialists, to provide integrated services in addition to their role with mental healthcare multidisciplinary teams, the aim being to deliver consistent practice and provide an essential and responsive service to acute and vulnerable cases during the period of hospitalisation.^5 10 11^ Social Work England has reported the number of social workers who registered from 2021 to 2022, classified by area of practice. Those who registered to work in adult mental health were 8710 (8.7%), and those in children’s mental health were 2170 (2.2%).^2^

Despite this long history of involvement, studies have highlighted that social workers face difficulties clarifying and justifying their contributions to mental healthcare,^4^ and that the social worker’s role and value remain unclear among mental health professionals.^11^,^13^ A recent scoping review, drawing on 35 qualitative studies, concluded that social workers faced significant challenges within mental healthcare, particularly in terms of developing and expressing their identity.^14^

One of the drawbacks is that quantitative research has been very limited on social worker contacts in routine mental healthcare. In part, this is because profession-specific contacts are not captured in national healthcare data resources. Consequently, service-wide evidence has been limited on social work involvement, and there is an impoverished data resource for quantifying and evaluating interventions in clinical practice. The first requirement is a means of ascertaining social work involvement in the first place. Using a large and granular mental health service data resource in south London, we sought to construct a dataset of social worker contacts in order to describe frequencies and distributions as a first step towards more in-depth evaluations of social work interventions and their outcomes.

## Methods

### Study setting and data source

A descriptive investigation of patient characteristics and circumstances associated with mental healthcare social worker contacts was conducted using data from the South London and Maudsley NHS Foundation Trust (SLaM). SLaM is one of the largest secondary mental healthcare providers in Europe, offering a comprehensive range of mental health services, both inpatient and outpatient/community-based, to a geographic catchment of around 1.3 million residents of four south London boroughs (Lambeth, Southwark, Lewisham and Croydon). A fully electronic health record (EHR) has been used in all SLaM services since 2006, and the Clinical Record Interactive Search (CRIS) was created in 2007–2008 to provide researchers with secure access to full data from over 500 000 de-identified health records within a robust governance framework.^15^,^17^ CRIS allows researchers to use any combination of data from structured and unstructured source fields to assemble data for secondary analysis; for this, data remain within a secure healthcare firewall for analysis, and a patient-chaired oversight committee oversees all usage.^15 18^ CRIS has received ethical approval as an anonymised data resource (Oxford Research Ethics Committee C, reference 23/SC/0257). The security model was approved in relation to national data governance law that authorises and governs the use of anonymised data for research purposes without specific consent, as CRIS adopts an opt-out model for data inclusion accompanied by a publicity programme to maximise awareness of the data platform among Trust service users.

### Sample

From all patients who received treatment in any services within SLaM from 2008 to 2023, using CRIS we sought to ascertain those who had at least one contact with a social worker and who were aged 16 years or over, based on case note entries describing clinical contacts.^19^ Characteristics and clinical status were ascertained at or as close as possible to the date of social worker contact.

### Distribution of social worker contacts

Social worker contacts were ascertained from the ‘Professional Group ID’ associated with generic forms completed for patient contacts in the source record. They were initially described by time and recorded modality of contact. For descriptive analyses of the sample characteristics at the level of the social worker contact event, we obtained the following patient information at or closest in time to that date: age, gender, ethnicity, marital status, Index of Multiple Deprivation and diagnosis. The IMD is the official measurement in England (2019) for the deprivation of relatively small areas/neighbourhoods, ascertained here at the level of the Lower Super Output Area, a standard administrative unit with around 1500 residents.^20^ The IMD score is based on a number of factors aggregated for that geographic unit from national census data (income, employment, education, health, crime, barriers to housing and services, living environment) and was assigned for the recorded address closest to the contact event.^20^ Regarding diagnoses, these are typically provided by the clinical team member in charge of the case through a team discussion^15^ and are recorded in prestructured fields according to codes from the International Classification of Diseases 10th Edition (ICD-10).

### Patient and public involvement

At the project planning stage, members of a Service User Advisory Group were shown initial ideas and plans and asked for thoughts and suggestions relating to a broader body of work (a PhD programme for NA) of which this study was a component. The group consisted of service users (of SLaM services) with lived experience of mental health problems. They were very keen on finding out the results of the first author’s PhD study. Suggestions were taken on board and informed, in part, some of the research questions.

## Results

Of the 260 844 total number of patients aged 16+ years under the care of SLaM between 2008 and 2023, 20.3% had at least one recorded attended event with a social worker. Considering all contacts, attended or not, 1 541 078 events involving a social worker were ascertained for 101 117 patients. Table 1 summarises the distribution of these contacts by patient demographic characteristics with illustrative comparison characteristics, previously published from the CRIS data resource of patients receiving active care (n=31 961) on a 31 December 2014 census date.^17^ Attended social worker contacts were most frequently in the 20–39 years age group, were more likely in males than females, were most common in patients of white followed by black ethnicity and in non-cohabiting patients. Considering previously published CRIS sample characteristics, the social work contacts were over-represented in the 20–59 years age groups, in black and Asian ethnic groups (although ethnicity was less likely to be missing) and in non-cohabiting patients. The majority of social work contacts occurred in patients living in neighbourhoods within the most deprived national quintiles. Attended social work contacts tended to be similar to the total contacts, except that those who attended were more likely in older patients and in those from less deprived neighbourhoods.

**Table 1:** Descriptive data for the total ascertained social work contacts (n=1 541 078) and the confirmed attended events (n=1 200 580) compared with previously published characteristics of all SLaM patients receiving active care (n=31 961) on 31 December 2014 census date^17^

Characteristic	Total events (%)*	Attended events (%)*	Comparison characteristics on 31 December 2014 (%)*
Age at event (years)			
<20	227 235 (14.7)	132 867 (11.1)	19.6
20–39	587 311 (38.1)	473 172 (39.4)	29.6
40–59	539 352 (35.0)	441 075 (36.7)	31.6
60–79	150 267 (9.7)	122 717 (10.2)	12.6
≥80	36 667 (2.3)	30 665 (2.6)	6.6
Gender*			
Female	738 943 (48.0)	568 235 (47.3)	47.5
Male	799 047 (51.9)	630 361 (52.5)	52.5
Ethnicity*			
White	679 624 (45.3)	530 821 (45.3)	60.1
Mixed	77 614 (5.1)	54 705 (4.7)	3.9
Asian	94 281 (6.2)	74 477 (6.4)	4.5
Black	582 554 (38.8)	461 965 (39.5)	24.8
Other	64 807 (4.3)	48 948 (4.2)	6.7
*Missing*	*42 198* (*2.7*)	*29 664* (*2.5*)	*8.8*
Marital status*			
Cohabiting	134 527 (10.0)	111 654 (10.4)	10.9
Non-cohabiting	1 208 697 (89.9)	958 207 (89.6)	77.4
Missing or not disclosed	197 853 (*12.8*)	*130 719* (*10.9*)	*11.7*
National deprivation quintiles			
1 (most deprived)	572 629 (39.3)	276 033 (26.73)	
2	579 967 (39.8)	240 881 (23.33)	
3	232 972 (16.0)	239 013 (23.15)	
4	56 673 (3.9)	241 254 (23.37)	
5 (least deprived)	16 298 (1.1)	35 318 (3.42)	

* Missing data not included in percentages.

**Figure 1:**
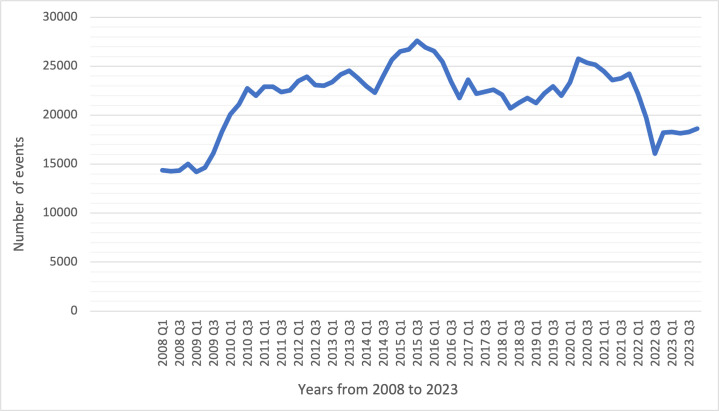
The line chart shows the distribution of the number of social workers’ contacts by years in SLaM services. SLaM, South London and Maudsley NHS Foundation Trust.

Figure 1 shows the trends in social work contacts over the ascertainment period from 2008 to 2023. The number of events increased sizeably from 2009 Q1 (n=14 202) to 2010 Q3 (n=22 755). Subsequently, the rates fluctuated until a further increase from 2014 Q2 (n=22 304) to 2015 Q3 (n=27 588), followed by a decline to 2018 Q2 (n=20 692), an increase to 2020 Q3 (n=25 341), and then a substantial decline to stabilise at 18 611 events during 2023 Q4.

**Figure 2:**
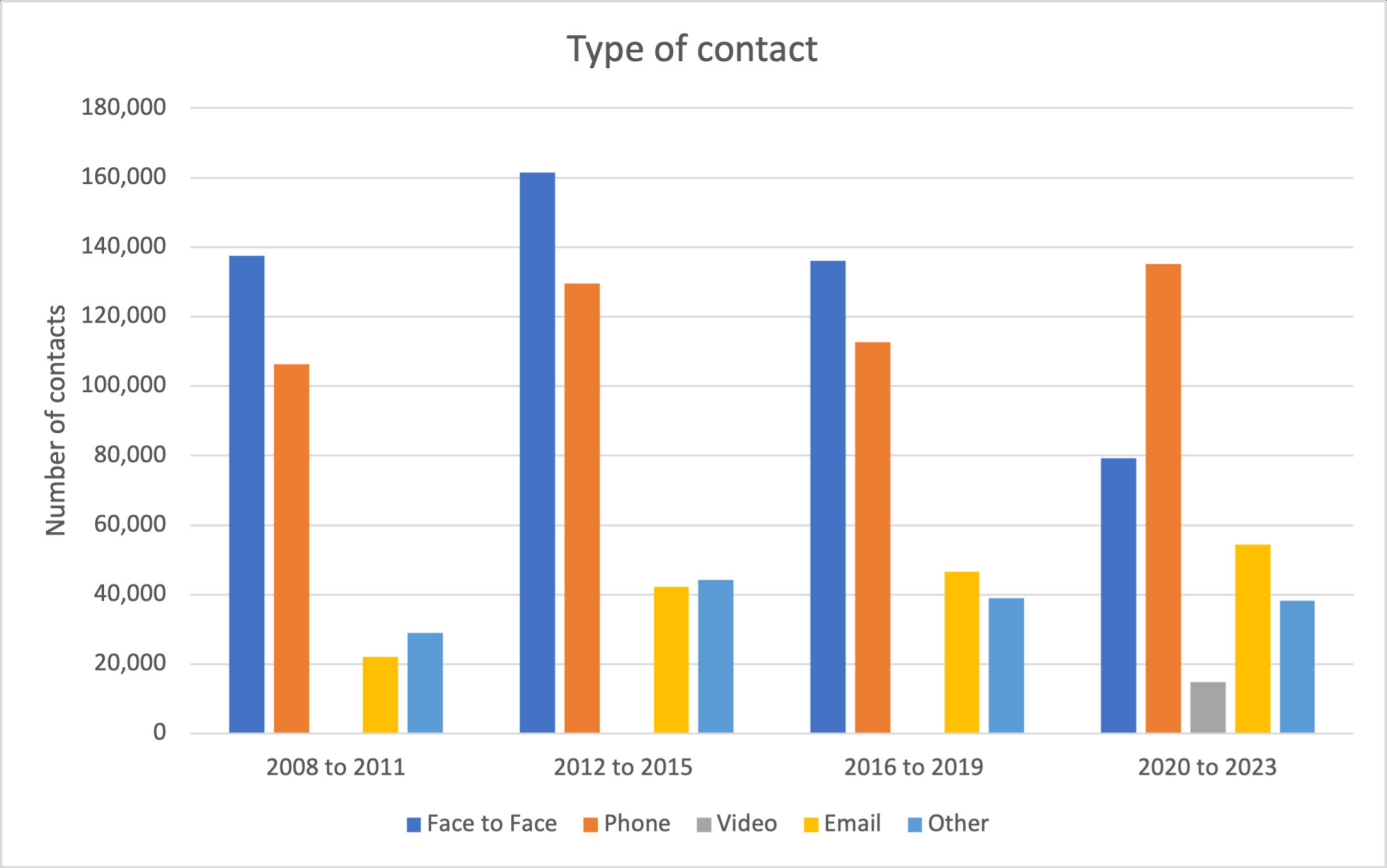
The bar graph shows the distribution of the number of contacts by different types of contacts from 2008 to 2023.

Figure 2 further describes the type of ascertained contact over the same period; face-to-face was the most common type of contact from 2008 to 2019. In 2020–2023, the phone was the most common type of contact used in addition to the online contact option that was implemented during the COVID-19 pandemic.

Table 2 summarises the distribution of types of contact by patient demographic status. Face-to-face contacts were more likely in younger patients, in males, in black compared with white ethnic groups, in patients who were not cohabiting and in patients from more deprived neighbourhoods. Significant associations were found between face-to-face contact (vs all other modalities) and all demographic characteristics.

**Table 2:** Types of social worker contact and distribution by patient characteristics (n=1 471 700).

Characteristic	Type of contact (%*)					Face to face vs non-face to face χ^2^ (P value)
	**Face to Face**	**Phone**	**Video**	**Email**	**Other**	
	(**n=5 82 816**)	(**n=5 39 602**)	(**n=14 921**)	(**n=1 72 185**)	(**n=1 62 176**)	
Age (years)						5468.2 (<0.001)
<20	12.3	11.9	6.9	13.6	11.0	
20–39	38.6	40.0	50.6	36.9	40.2	
40–59	38.3	34.4	30.6	34.9	35.7	
60–79	9.2	10.4	10.1	11.6	10.3	
≥80	1.5	3.2	1.9	3.0	2.7	
Gender						2781.4 (<0.001)
Female	44.9	50.1	51.8	48.3	47.3	
Male	55.0	49.7	47.7	51.4	52.5	
Ethnicity						1985.3 (<0.001)
White	43.0	45.1	42.2	44.7	44.2	
Mixed	4.5	4.8	7.0	5.2	4.8	
Asian	6.2	6.1	5.6	6.1	5.8	
Black	40.6	37.1	35.0	35.4	36.0	
Other	3.9	4.1	4.2	4.9	4.9	
*Missing*	*1.8*	*2.8*	*5.9*	*3.7*	*4.2*	
Marital status						262.2 (<0.001)
Cohabiting	8.7	9.5	7.6	8.0	9.0	
Non-cohabiting	81.9	78.8	61.7	77.2	76.3	
*Missing or not disclosed*	*9.4*	*11.7*	*30.7*	*14.8*	*14.6*	
National deprivation quintiles						897.3 (< 0.001)
1 (most deprived)	27.7	27.2	28.0	27.2	27.9	
2	48.7	47.3	44.2	46.1	46.8	
3	18.3	19.4	19.8	19.9	19.0	
4	4.3	4.7	6.0	5.1	4.7	
5 (least deprived)	1.1	1.4	2.0	1.6	1.5	

Social worker contacts are additionally described by closest primary diagnosis in table 3 grouped according to ICD-10 chapters. Contacts occurred much more frequently in patients with schizophrenia and related disorders (39.2%) than any other diagnosis, followed by mood disorder (16.0%) and unspecified mental disorder (7.8%). As a comparison, in the 2014 CRIS-wide census reported that of patients receiving active SLaM care, 21.1% had ever received a diagnosis of schizophrenia or related disorders (ICD-10 F2x), 19.0% had ever received a mood disorder (F3x) diagnosis and 22.0% had ever received an unspecified mental disorder diagnosis (F99).^17^

**Table 3:** Distribution of social worker contacts by recorded primary diagnosis

Primary diagnosis (ICD-10 code and description)	Number of contacts
F00–F09 Organic, including symptomatic, mental disorders	63 038 (4.1)
F10–F19 Mental and behavioural disorders due to psychoactive substance use	62 851 (4.1)
F20–F29 Schizophrenia, schizotypal and delusional disorders	603 387 (39.2)
F30–F39 Mood (affective) disorders	246,22 (16.0)
F40–F48 Neurotic, stress-related and somatoform disorders	95 446 (6.2)
F50–F59 Behavioural syndromes associated with physiological disturbances and physical factors	13 671 (0.9)
F60–F69 Disorders of adult personality and behaviour	102 491 (6.7)
F70–F79 Mental retardation	4024 (0.3)
F80–F89 Disorders of psychological development	25 994 (1.7)
F90–F98 Behavioural and emotional disorders with onset usually occurring in childhood and adolescence	67 592 (4.4)
F99–F99 Unspecified mental disorder	120 186 (7.8)
Z00–Z99 Factors influencing health status and contact with health services	83 257 (5.4)

ICD-10International Classification of Diseases 10th Edition

To describe the patients’ characteristics in more detail in relation to the level of social worker contact, we selected one calendar year from 1 January to 31 December 2016 and compared events that were occurring fewer than 20 times per patient in that year with those occurring 20 times or more. Tables4  5 illustrate the patient characteristics of frequent and less frequent events. Frequent events were over-represented in the <20 years and 40–59 years age groups, marginally in women compared with men, in non-cohabiting patients, in black and mixed ethnic groups and in higher deprivation neighbourhoods (table 4). Diagnostically, they were over-represented in schizophrenia and related disorders, personality disorders and child/adolescent disorders (table 5).

**Table 4:** Characteristics of the distribution of SLaM patients based on the frequency of recorded events from 1 January to 31 December 2016.

Total number of events (97 175)	Frequent event*(n=44 141)	Infrequent event†(n=53 034)
Age (years)		
<20	6749 (15.3)	6639 (12.5)
20–39	14 406 (32.6)	19 396 (36.6)
40–59	18 421 (41.7)	20 201 (38.1)
60–79	4512 (10.2)	5463 (10.3)
≥80	53 (0.1)	1326 (2.5)
Gender		
Female	21 411 (48.5)	25 632 (48.3)
Male	22 590 (51.2)	27 364 (51.6)
Marital status^‡^		
Cohabiting	2803 (6.8)	5751 (12.2)
Non-cohabiting	38 407 (93.2)	41 561 (87.8)
Missing or not disclosed	*2931* (*6.6*)	*5722* (*10.8*)
Ethnicity^‡^		
Asian	2121 (4.8)	3531 (7.0)
Black	17 311 (39.5)	18 299 (36.1)
Mixed	2433 (5.6)	2145 (4.2)
Other	1912 (4.3)	2872 (5.7)
White	20.002 (45.7)	23 861 (47.1)
Missing	*362* (*0.8*)	*2326* (*4.4*)
National deprivation quintiles		
1 (most deprived)	23 367 (52.9)	25 221 (47.6)
2	6792 (15.4)	9404 (17.7)
3	3428 (7.8)	4671 (8.8)
4	2224 (5.0)	2634 (5.0)
5 (least deprived)	8329 (18.9)	11 105 (20.9)
	Number of patients=13 392	

* More than 20 events recorded.

† Fewer than 20 events recorded.

‡ Missing data not included in percentages.

SLaMSouth London and Maudsley NHS Foundation Trust

**Table 5:** Distribution of frequent and infrequent social worker contacts by recorded primary diagnosis

Primary diagnosis (ICD-10 code and description)	Frequent*	Infrequent†
F00–F09 Organic, including symptomatic, mental disorders	533 (1.2)	2154 (4.2)
F10–F19 Mental and behavioural disorders due to psychoactive substance use	1816 (4.2)	3149 (6.1)
F20–F29 Schizophrenia, schizotypal and delusional disorders	18 616 (42.9)	16 966 (32.9)
F30–F39 Mood (affective) disorders	7208 (16.6)	8696 (16.9)
F40–F48 Neurotic, stress-related and somatoform disorders	2556 (5.9)	3943 (7.7)
F50–F59 Behavioural syndromes associated with physiological disturbances and physical factors	150 (0.3)	523 (1.0)
F60–F69 Disorders of adult personality and behaviour	5372 (12.4)	3062 (5.9)
F70–F79 Mental retardation	61 (0.1)	123 (0.2)
F80–F89 Disorders of psychological development	593 (1.4)	908 (1.8)
F90–F98 Behavioural and emotional disorders with onset usually occurring in childhood and adolescence	2036 (4.7)	1745 (3.4)
F99–F99 Unspecified mental disorder	2751 (6.3)	7032 (13.6)
Z00–Z99 Factors influencing health status and contact with health services	1702 (3.9)	3220 (6.2)

* More than 20 events recorded.

† Fewer than 20 events recorded.

ICD-10International Classification of Diseases 10th Edition

## Discussion

The broad purpose of this research was to build capacity for investigations of social work intervention in mental healthcare using large, routine data resources. As a first step to achieve this, we used the available anonymous source to present descriptive data on the characteristics of patients who received contact from a social worker as a component of their mental healthcare. Our findings showed that contacts were predominantly in working age groups, and men were slightly more represented than women. A relatively high number of contacts occurred in patients from black ethnic groups—more than would be expected from previously reported patient distributions. The largest numbers were also in patients with diagnosed schizophrenia and related disorders. More frequent events were over-represented in non-cohabiting patients, in black and mixed ethnic groups, higher deprivation neighbourhoods and particular diagnostic groups (schizophrenia and related disorders, personality disorders, child/adolescent disorders), likely consistent with higher social need. Over the 16 years evaluated, the number of social worker contacts peaked in 2015, which could be influenced by the Care Act 2014 and adult safeguarding considerations.^21^

Information on the distribution of social worker contacts has been limited in the existing literature to date for the reasons outlined earlier. However, in an early study, researchers conducted a comparison between nurses and social workers in community mental health services, analysing 283 nurse and 78 social worker contacts. Considering distributions between diagnoses, of nurse contacts 54% were with patients diagnosed with schizophrenia, and 33% were with depression and/or anxiety.^22^ In comparison, of social worker contacts, 41% were with patients with depression and/or anxiety, and 38% were with schizophrenia. Social workers were also found to spend more time with patients, an average of 25 min per individual. More recently, a study included a secondary analysis of the Community Mental Health Service User Survey from the Care Quality Commission (2016); the sample for that analysis was 2575 patients coordinated by nurses and 682 by social workers and, with both, female service users 66 years old and over were the highest represented group.^23^

As previously described, there has been very little published evidence on social work involvement and deployment in mental healthcare to date. A systematic review was conducted in 2022 about psychosis in the social work literature which identified only nine studies. The results of this review highlighted the lack of literature on psychotic disorders in most social work journals and suggested that clinical work in the profession might be impeding research interests.^24^ Social interventions in psychosis have received more attention. For example, a 3-year prospective study investigated social support and clinical outcomes for 152 people diagnosed with schizophrenia receiving community mental health input, finding positive influences on patients’ clinical and functional outcomes.^25^

While there are limited publications on social work involvement using routine mental healthcare data, there is a broader literature on the nature of involvement using small samples and in-depth qualitative evidence. For example, in mixed-method studies of social work role evaluation in community mental health services, Abendstern and colleagues administered semistructured interviews for 42 staff members from multiple disciplines working in CMHTs with older people, only four of whom were social workers.^2 26^ However, a larger number of studies have investigated social workers’ perspectives in mental healthcare. Focusing on the social work voice and their colleagues emphasised challenges faced and the importance of supporting contributions as specialists in the integrated system.^1^ In another report, the authors followed up their national survey of community mental health teams in a large mixed methods study^27^,^29^; in that analysis, the participants were 188 team managers who filled out the questionnaire from 50 English Trusts in 2018, and the report highlighted that the social work role in community mental health teams is unstable, and that managers value social work involvement and are apprehensive that this instability may affect the profession’s future. In other contexts, the importance of mental health social workers has been reiterated for acute hospital settings^10^ and has been considered in relation to suicide prevention^30^ and women’s mental health.^31^ Therefore, a need has been articulated for investigating social workers’ involvement in mental healthcare. Using routine mental healthcare data provides a mechanism to generate quantitative and potentially replicable investigations and hence the rationale for the approach we have taken here.

The key strength of our study is that it opens up a route towards a better understanding of the application of social work as a discipline in routine mental healthcare, more specifically providing what we believe to be the first descriptive data on the distribution of contacts over time and by patient characteristics. The large and long-term data enable these descriptions. Moreover, these data are from routine real-world clinical practice, which can be combined with rich further information, both within CRIS and from its range of linked external data resources,^17 32 33^ for developing knowledge about social workers’ involvement and role in the mental healthcare system. However, there are important limitations that also need to be considered. Despite the underlying diversity in SLaM’s services and catchment population, the data in this study arise from a specific urban and semi-urban geography. The comparison population was by necessity a convenience sample, in which the full CRIS population had been characterised on 31 December 2014, approximately midway through the evaluation period for our study. CRIS is a live database, and the population represented on the platform increases by around 20 000 per annum. While there has been no evidence from past CRIS research of substantial changes in the sociodemographic characteristics of patients receiving services over time, comparisons can only be viewed as illustrative approximations. Additional limitations are generic to routine administrative data that are not collected specifically for research, such as the level of missing data on some variables, and the potential for error. We did not seek to classify or categorise the intervention or assessment being delivered via the social work contact, which will require further code development if it is to be achieved at scale across EHR data resources—for example, to distinguish Mental Health Act, safeguarding and other roles. Comparisons by modality of contact might reflect temporal variations, as there were substantial changes in contact modality during the COVID-19 pandemic in 2020–2021. Finally, it should be borne in mind that the focus here was on capturing input from social workers who are part of mental health service teams, and we did not seek to capture input from those employed externally—this is likely to account for the relatively low representation of contacts in younger age groups or in patients with dementia, where social work provision is most likely to be external from Local Authorities.

In conclusion, we believe we describe a unique and novel approach to investigating social work involvement within mental healthcare. Descriptive data have been successfully generated on the characteristics of patients receiving these contacts, opening up opportunities for future studies to investigate health outcomes and create an evidence base for formulating and evaluating policy.

## Data Availability

No data are available.
