# Supplementary material for: Ascertaining social worker contacts in routine mental healthcare and describing their distribution: a descriptive analysis of electronic records data from a large south London mental healthcare provider
Source: BMJ Open. 2025 Mar 26;15(3):e090055. doi: 10.1136/bmjopen-2024-090055 (PMC11950943; doi:10.1136/bmjopen-2024-090055)
Supplement: Supplementary file 1 [file bmjopen-2024-090055.reviewer_comments.pdf]

PEER REVIEW HISTORY

BMJ Open publishes all reviews undertaken for accepted manuscripts. Reviewers are asked to complete a checklist review form and are provided with free text boxes to elaborate on their assessment. These free text comments are reproduced below.

ARTICLE DETAILS

Title (Provisional)

Ascertaining social worker contacts in routine mental healthcare and describing their distribution: A descriptive analysis of electronic records data from a large south London mental healthcare provider.

Authors

Alothman, Norah; Jewell, Amelia; Perera, Gayan; Stewart, Robert

---

VERSION 1 - REVIEW

Reviewer **1**

Name **Clavenna, Antonio**

Affiliation **Istituto di Ricerche Farmacologiche Mario Negri, Laboratory for Mother and Child Health, Department of Public Health, Department of Public Health**

Date **19-Oct-2024**

COI **None**

Thank you for the possibility to review this paper.

I have some comments and suggestions that I hope may be helpful in improving your manuscript

1. Would it be possibile to have an estimate of the total number of people who attended SLaM in the period 2008-2023? In my opinion, it would be useful to provide the percentage of patients with at least one social worker contact.

In the case of figure 1: which is the amount of overall contacts by year?

2. You compared the characteristics of social worker contacts with those of CRIS at 31 December 2014. Are you confident that the characteristics of people receiving care did not change across time?

3. Regarding the comparison of type of contact (Table 2): some differences may be related to the decrease of face-to-face contact in the post-covid period. Moreover, it could be helpful to perform a chi-square test to evaluate the association between characteristics and face-to-face contact (yes/no).

4. It seems to me that the discussion is mainly focused on the involvement of social workers in mental healthcare and in some parts not really pertinent to the findings of the study (for example: page 14, lines 16-44)

**Reviewer**	**2**
**Name**	**Dennis, Brittany**
**Affiliation**	**The University of British Columbia, Medicine**
**Date**	**07-Nov-2024**
**COI**	**None**

The aim of this study was to describe the distribution of contacts with mental health service social workers between 2008 and 2023 and by patient characteristics in a sample of UK patients accessing a large secondary healthcare provider in London UK. The purpose of this study was to describe the population of patients accessing mental health social workers. In general, the researchers found patients accessing social workers were often young (20-39), white men, who live alone, in neighbourhoods with lower socioeconomic status. In general schizophrenia was over-represented. The purpose of this work is to help elucidate the nature of social work deployment among patients seeking mental health services.

The authors conducted an evaluation of social work deployment trends employing administrative data, a de-identified database of over 500,000 health records from the South London and Maudsley NHS Foundation Trust (SLaM). The data presented from this study highlights a signal demonstrating the types of patients more likely to engage with community social work services, in addition to interesting gender-based differences in the types of engagement (in person, telephone) patients may prefer.

Despite the immense work undertaken in this study, it is not without some limitations. Overall, the authors findings should be interpreted within the context of the data, which reflects the characteristics of patients who made connection with a social worker, a population which may differ greatly from those unable to engage. Given the purpose of this work is to better characterize social work deployment trends, this must be considered given there may be a large degree of social worker deployment not captured in this data.

I will summarize my concerns and suggestions below

1) Methods

a. The authors relied on administrative data, specifically de-identified health records of patients receiving care at a south London NHS trust. I am a little concerned with the case definition, namely the reliance on identifying only those patients who had at least one contact with a social worker. Within this definition, you would not capture patients who were unable to engage with the social worker (who often may be the most vulnerable and high-risk population). Thus, you would be unable to truly characterize the breadth of social work deployment. Is there any way to capture in the database those patients who have had a social work consult placed, but not fulfilled? If not, the implications of this should just be discussed more in the limitations

b. The authors have chosen to describe the characteristics of the general population of patients in contact with a social worker. Is there ability to disaggregate this data further based of degree of social work contact. I think a description of the patient population with the higher

burden of social work demands (i.e., 3-4 contacts per week) would be a relevant and important finding to better understand where social work capacity is being most used and in what populations further focus/resources could be targeted

2) Results and Interpretation: The results are summarized clearly and concisely. I have no concerns with how the data are represented in the results section.

The authors have provided readers with an in-depth evaluation of social work deployment trends among patients seeking mental health services in the South London region. Overall, this work is important, relevant, and stands to inform public policy, monitoring, and future studies aimed at exploring ways to optimize resource allocation to better support patients struggling with mental illness in community settings.

VERSION 1 - AUTHOR RESPONSE

Reviewer (1):

1. **Would it be possible to have an estimate of the total number of people who attended SLaM in the period 2008-2023? In my opinion, it would be useful to provide the percentage of patients with at least one social worker contact. In the case of figure 1: which is the amount of overall contacts by year?**

Thank you for your comments. In response, we have requested and added the required information in the results section. We have also added a y-axis label in Figure 1.

Of the 260,844 total number of patients aged 16+ under the care of SLaM between 2008-2023, 20.3% had at least one recorded attended event with a social worker.

2. **You compared the characteristics of social worker contacts with those of CRIS at 31 December 2014. Are you confident that the characteristics of people receiving care did not change across time?**

Thank you for your comment. We have added the following statement in the discussion, acknowledging that this comparison was illustrative and approximate, although we do not believe there are major changes in characteristics over time to account for any observed differences.

The comparison population was by necessity a convenience sample, in which the full CRIS population had been characterised on 31st Dec 2014, approximately midway through the evaluation period for our study. CRIS is a live database, and the population represented on the platform increases by around 20,000 per annum. While there has been no evidence from

past CRIS research of substantial changes in the sociodemographic characteristics of patients receiving services over time, comparisons can only be viewed as illustrative approximations.

3. **Regarding the comparison of type of contact (Table 2): some differences may be related to the decrease of face-to-face contact in the post-covid period. Moreover, it could be helpful to perform a chi-square test to evaluate the association between characteristics and face-to-face contact (yes/no).**

Thank you for bringing this to our attention. We have added text to the limitations section to acknowledge the issue of changes in contact modality during the COVID-19 pandemic.

The comparison population was by necessity a convenience sample, in which the full CRIS population had been characterised on 31st Dec 2014, approximately midway through the evaluation period for our study. CRIS is a live database, and the population represented on the platform increases by around 20,000 per annum. While there has been no evidence from past CRIS research of substantial changes in the sociodemographic characteristics of patients receiving services over time, comparisons can only be viewed as illustrative approximations.

As requested, we have added chi-squared tests to evaluate the association between characteristics and face-to-face contact in Table 2, and these findings are described in more detail in the Results section:

Table 2 summarises the distribution of types of contact by patient demographic status. Face-to-face contacts were more likely in younger patients, in males, in Black compared to White ethnic groups, in patients were were not cohabiting, and in patients from more deprived neighbourhoods. Significant associations were found between face-to-face contact (vs. all other modalities) and all demographic characteristics.

4. **It seems to me that the discussion is mainly focused on the involvement of social workers in mental healthcare and in some parts not really pertinent to the findings of the study (for example: page 14, lines 16-44).**

Thanks for your comments. We have altered the text around this paragraph to try to make the contextual relevance clearer. In the absence of directly relevant evidence in the literature (as far as we could find), we felt that it was importance to acknowledge at least

some evidence on social worker involvement which we believe is complementary to our findings in terms of the broader evidence base, even if not directly relevant. We have tried to make this clearer.

Reviewer: 2

a. **The authors relied on administrative data, specifically de-identified health records of patients receiving care at a south London NHS trust. I am a little concerned with the case definition, namely the reliance on identifying only those patients who had at least one contact with a social worker. Within this definition, you would not capture patients who were unable to engage with the social worker (who often may be the most vulnerable and high-risk population). Thus, you would be unable to truly characterize the breadth of social work deployment. Is there any way to capture in the database those patients who have had a social work consult placed, but not fulfilled? If not, the implications of this should just be discussed more in the limitations**

Thank you for pointing this out. In response to this comment, we have included an additional column to Table 1 so that this now displays all events and those specifically attended. Additional text has been provided in the Results to cover this.

Attended social work contacts tended to be similar to the total attempted contacts, except that those attended were more likely in older patients and in those from less deprived neighbourhoods.

b. **The authors have chosen to describe the characteristics of the general population of patients in contact with a social worker. Is there ability to disaggregate this data further based of degree of social work contact. I think a description of the patient population with the higher burden of social work demands (i.e., 3-4 contacts per week) would be a relevant and important finding to better understand where social work capacity is being most used and in what populations further focus/resources could be targeted**

Thank you for raising this point. Because all the analyses in this paper are at the level of the event rather than of the patient, we felt that it would be problematic attempting a patient-level comparison, which would have required a new sample and methodology. Instead, we sought to address this comment by selecting one calendar year from 1st January to 31st December 2016 and to describe event-level characteristics for events which were or were not

frequently repeated in the same patients in that period. The findings are presented in newly added Tables 4 and 5 and are cited in the Results and Discussion.

In order to describe the patients' characteristics in more detail in relation to the level of social worker contact, we selected one calendar year from 1st January to 31st December 2016 and compared events which were occurring fewer than 20 times per patient in that year with those occurring 20 times or more. Tables 4 and 5 illustrate the patient characteristics of frequent and less frequent events. Frequent events were over-represented in the <20 and 40-59 age groups, in women compared to men, in non-cohabiting patients, in Black and Mixed ethnic groups, and in higher deprivation neighbourhoods (Table 4). Diagnostically, they were over-represented in schizophrenia and related disorders, personality disorders, and in child/adolescent disorders (Table 5). More frequent events were over-represented in non-cohabiting patients, in Black and Mixed ethnic groups, higher deprivation neighbourhoods and particular diagnostic groups (schizophrenia and related disorders, personality disorders, child/adolescent disorders), likely consistent with higher social need.

VERSION 2 - REVIEW

Reviewer **1**

Name **Clavenna, Antonio**

Affiliation **Istituto di Ricerche Farmacologiche Mario Negri, Laboratory for Mother and Child Health, Department of Public Health, Department of Public Health**

Date **24-Feb-2025**

COI

In my opnion, comments and suggestions by the reviewers were addressed in a satisfactory manner

---

Reviewer **2**

Name **Dennis, Brittany**

Affiliation **The University of British Columbia, Medicine**

Date **05-Mar-2025**

COI

No further concerns. Issues addressed.
